# Characteristics of lactic acid bacteria, microbial community and fermentation dynamics of native grass silage prepared in Inner Mongolian Plateau

**DOI:** 10.3389/fmicb.2022.1072140

**Published:** 2023-01-09

**Authors:** Meiling Hou, Zhijun Wang, Lin Sun, Yushan Jia, Shicong Wang, Yimin Cai

**Affiliations:** ^1^College of Life Science, Baicheng Normal University, Baicheng, China; ^2^Key Laboratory of Forage Cultivation, Processing and High Efficient Utilization of Ministry of Agriculture, Key Laboratory of Grassland Resources, Ministry of Education, College of Grassland, Resources and Environment, Inner Mongolia Agricultural University, Hohhot, China; ^3^Inner Mongolia Academy of Agricultural Science & Animal Husbandry, Hohhot, China; ^4^Japan International Research Center for Agricultural Sciences (JIRCAS), Tsukuba, Japan

**Keywords:** 16S rRNA gene, native grass, lactic acid bacteria, silage fermentation, isolation and identification

## Abstract

**Introduction:**

To prepare high-quality silage, we studied the chemical composition, silage fermentation, characterization, and identification of lactic acid bacteria (LAB) associated with the silage fermentation of native grass on the Inner Mongolian Plateau.

**Methods:**

LAB were isolated from fresh native grass and their silage, and silages were prepared using a small-scale fermentation system with 2–3 cm length in plastic bags.

**Results:**

The dominant species of native grasses used were *Stipa baicalensis*, *Leymus chinensis*, *Cleistogenes squarrosa*, *Melissilus ruthenicus* and *Pulsatilla turczaninovii*, which contained 47.83–59.43 % moisture, 55.12–67.74 % neutral detergent fiber (NDF), and 8.72–14.55 crude protein (CP), and these nutrients did not change greatly during ensiling. Good preservation with a relatively low pH (below 4.44) and high (*p* < 0.05) lactic acid content (>0.58) was obtained after ensiling. Based on the morphological and biochemical characteristics, these isolates were divided into 12 groups (A-L). All isolate strains were gram-positive and catalase-negative bacteria that produce lactic acid from glucose. Group A-K were cocci, while group L was rod-shaped. Group A-E formed D-lactic acid, but group H-K formed L-lactic acid, and other groups formed DL-lactic acid. Group A-E were heterofermentative, and Group F-L were homofermentative types of LAB. According to the 16S rRNA gene sequences analysis, strains were identified as genus *Leuconostoc* (A, B, and C), *Weissellla* (D, E), *Pediococcus* (F, G), *Enterococcus* (H, I, J and K), and *Lactiplantibacillus* (L). *Enterococcus* (E.) *faecium* (29.17%, percentage of total isolates) and *Pediococcus (P.) acidilactici* (18.75%) were the most frequently occurring dominant species.

**Discussion:**

This study suggests that the native grasses contained abundant LAB species, and they can be used as good-quality silages in animal husbandry. In addition, the strains *P. acidilactici* and *E. faecium* were the most frequently isolated from native grass silages as dominant species which can be a potentially excellent inoculant for native grass silage.

## 1. Introduction

Alongside hay, native grass is regarded as an important resource for animal feed in the Mongolian Plateau, and a large quantity is produced annually in pastoral areas (Hou et al., 2017; Ge et al., 2018; You et al., 2021b). Grazing in native grasslands during summer/autumn and feeding hay during winter/early spring are traditional methods of animal rearing in this region, and these native grasses support the local livestock by providing roughage supplement throughout the year (Bu et al., 2021; Du et al., 2022). However, the harvest is always carried out in early autumn, and since weather is unpredictable, silage production has become a better way to preserve native grass than hay-making, which also allows for long-term storage of fresh grass, making it available throughout the year (Bu et al., 2021; Li Y. et al., 2022). Microorganisms including *Lactobacilli, Bacilli, Clostridia*, acetic acid bacteria, molds, and yeasts in preservation forages influence silage quality during ensiling. Studies have been conducted on microbe population and silage fermentation for ensiled corn, king grass, Italian ryegrass, and alfalfa (Pang et al., 2011; Shah et al., 2017; Yan et al., 2019; Wang et al., 2022). These showed that selected isolates from natural lactic acid bacteria (LAB) have the potential to improve silage quality and fermentation while inhibiting the growth of undesirable microbes under anaerobic conditions. Silage fermentation and their associated microbe communities have been studied using various microbiological techniques, such as 16S rRNA gene, DNA-DNA hybridization, and third-generation Pacific Biosciences (PacBio) single molecule real-time sequencing technology (SMRT), which generates long reads (Ennahar et al., 2003; Lee et al., 2018; Du et al., 2021). The phylogenetic relationships of LAB have been explored extensively in 16S rRNA sequence ribotyping and experiments, resulting in some new species, such as *Lactiplantibacillus nasuensis* sp. nov., *Lactococcus uvarum* sp. nov., *Lactiplantibacillus terrae* sp. nov., *Lactiplantibacillus xiangfangensis* sp. nov. isolated from silage, kimchi, milk, oil, and pickle being included in the microbe community.

High-quality silage from native grasses is generally difficult due to low water-soluble carbohydrate (WSC) content and higher buffering capacity (Hou et al., 2017). Certain natural LABs from native grasses play a vital role in fermentation (Ge et al., 2018). Further, the Mongolian Plateau might have special microorganisms regardless of water availability due to its frigid weather and dry climate (Zhang et al., 2015). Thus, collecting, screening, and identifying the LAB biochemically from native grass is important. In addition, some of the selected LAB might be potential well-sourced silage additions for forages.

## 2. Materials and methods

### 2.1. Silage preparation

Samples of native grass [Baical needlegrass (*Stipa baicalensis*), China leymus (*Leymus chinensis*), Scabrous hide seed grass (*Cleistogenes squarrosa*), Ruthenia medic (*Melissilus ruthenicus*), and Slenderleaf pulsatilla (*Pulsatilla turczaninovii*)] were collected from the meadow steppe of Hulunbuir (48.27°N, 119.44°E; Inner Mongolia, China) on 25 July 2019 (full-bloom stage). Before ensiling, the moisture of native grasses ranged from 48.34 to 59.43% FM. The grasses were cut into 3–5 cm lengths by using a chopper machine (DD-ZCD, Dedong Co., Ltd., Weifang, China), and the grass silages were prepared in the form of mini silos (polyethylene, 1L, Yijia, Beijing, China) without witling (density, 450–500 kg/m^3^). The silos were kept at ambient temperature (20–26°C). Five silos were used per treatment. After 30 days, the microbial community, chemical composition, and fermentation quality were analyzed.

### 2.2. Microbial population, chemical composition, and silage fermentation

Referring to Cai (1999), 10 g of each sample was blended with 90 ml saline solution (0.85% NaCl) and serially diluted from 10^−1^ to 10^−6^. The microbial community of the native grass was analyzed *via* plate cultivation. The numbers of LAB were measured using de Man, Rogosa, Sharp (MRS) agar after serial dilutions and incubated at 37°C for 48 h in an anaerobic box (Oxoid AnaeroJar, AG0025A, Thermo Fisher Scientific Inc., Waltham, MA, United States). Aerobic bacteria were counted on Blue Light Broth agar (Nissui-Seiyaku Ltd., Tokyo, Japan). Yeast and mold were counted on Potato Dextrose Agar (Oxoid, CM0139B, Thermo Fisher Scientific Inc., Waltham, MA, United States) and incubated at 37°C for 48 h. Yeasts were distinguished from mold and bacteria by colony appearance and observation of cell morphology. The coliform bacteria were counted on Blue Light Broth Agar (Nissui, Tokyo, Japan) at 37°C for 48 h. All microbial data were counted as viable numbers in colony-forming units (CFU) per gram on a fresh matter (FM) basis. For LAB isolation, 20 colonies of each sample were picked, 48 strains were collected and considered as LAB. The individual colonies were isolated and purified twice by steaking on MRS broth and Agar (Oxoid, CM1175/CM0003B, Thermo Fisher Scientific Inc., Waltham, MA, United States). Pure cultures were grown on MRS agar at 37°C for 24 h, resuspended in a solution of nutrient broth (Oxoid) containing 10% dimethyl sulfoxide, and stored at-80°C for further examination.

The dry matter (DM), organic matter (OM), crude protein (CP), and ether extract (EE) contents were measured according to the Association of Official Analytical Chemists (AOAC) methods (2005). The neutral (NDF) and acid (ADF) detergent fibers were obtained using the ANKOM A200i fiber analyzer (ANKOM Technology, Macedon, NY, United States) by Van Soest et al. (1991). The water-soluble carbohydrate (WSC) was determined by DNS colorimetric method (Sucrose/D-Glucose/D-Fructose UV-method, Roche Diagnostics, Tokyo, Japan) by Thomas (1997).

For silage fermentation analysis, another set of wet silage (10 g) samples was mixed with 90 ml of deionized water. The extracts were filtered through four layers of cheesecloth and filter paper (pore size 9 cm; Beimu, Hangzhou, China) and homogenized for 3 min. The pH was measured using a glass electrode pH meter (PHS-3E; LEICI, Shanghai, China). The NH_3_-N content was determined by the method of Broderick and Kang (1980) using steam distillation of the filtrates. The organic acid content was analyzed using the high-performance liquid chromatography (HPLC; GC-8A, Shimadzu, Kyoto, Japan) method (column: KC811, Shimadzu, Kyoto, Japan; detector: SPDM10AVP, Shimadzu, Kyoto, Japan; eluent: 3 mmol L^−1^ HCLO_4_, 1 ml min^−1^; temperature: 50°C) by Ennahar and Cai (2004).

### 2.3. Morphological, physiological, and biochemical tests

The Gram staining and observation of morphological characteristics of LAB were determined by MRS agar incubated at 37°C for 48 h (Zanoni et al., 1987). Catalase activity and glucose gas production were determined by the methods of Cai et al. (1999). Growth at different temperatures (5°C, 10°C, 45°C, and 50°C), different pH (2.5, 3.0, 3.5, 4.0, 7.0, and 9.0), and different salt solutions (3.0, 6.5% (w/v) NaCl) were determined on MRS agar at 30°C for 120 h. Carbohydrate fermentation of 49 different compounds was tested by Analytical Professorile Index (API 50 CH) strips (bioMerieux, Craponne, France) at 30°C for 24 h and verified for 48 h (Nomura et al., 1999).

### 2.4. Extraction of LAB genomic DNA and 16S rRNA gene sequence

Each strain was cultivated overnight in 5 ml MRS medium at 30°C, centrifuged at 9391 *g* for 5 min, washed twice with buffer (10 mmol L^−1^ Tris-HCl, 0.1 mmol L^−1^ EDTA, pH 8.0) in a clean 1.5 ml microcentrifuge tube, and recentrifuged. Genomic DNA was extracted through Plant Genomic DNA Kit (TIANGEN Biotech Co., Ltd., Beijing, China) according to the manufacturer’s instructions. The genomic DNA concentration of each strain was determined at 260 nm using a UV-VIS spectrophotometer (Shanghai Metash Instruments Co., Ltd., Shanghai, China; Zhang et al., 2016b). The extracted genomic DNA of each strain was stored at −20°C for future use.

For DNA extraction and purification, cells were grown at 37°C for 8 h (Akane et al., 1994), and the 16S ribosomal RNA (rRNA) gene sequences were amplified in a PCR Thermal Cycler (A48141, Thermo Fisher Scientific Inc., Waltham, MA, United States) by using the PCR and reagents from a Takara Taq PCR Kit (TIANGEN Biotech Co., Beijing, China). The sequences were determined directly with a sequencing kit (NOVA-5144, Bio Scientific Co., Ltd., Austin, United States) using the prokaryotic 16S rRNA universal primers 27F (5′-AGAGTTTGATCCTGGCTCAG-3′) and 1492R (5′-GGTTACCTTGTTACGACTT-3′) in combination with Applied Biosystems model 3500 (Applied Biosystems, Thermo Fisher Scientific Inc., Waltham, MA, United States) automated sequencing system (Cai, 1999). Sequence similarity searches were performed at the GenBank data library using the BLAST (Basic Local Alignment Search Tool) program.

### 2.5. Statistical analyses

The 16S rRNA sequences were constructed by the neighbor-joining method using Molecular Evolutionary Genetic Analysis (MEGA) 6.0 software (Saitou and Nei, 1987). Data on chemical composition and fermentation quality after 30 days were analyzed by one-way analysis of variance using the general linear model procedure of SAS version 9.3 (SAS Institute Inc., 2015). The differences between means were assessed using Turkey’s multiple comparison tests and were considered statistically significant at *p* < 0.05.

## 3. Results

### 3.1. Microbial parameters of native grass silages

The counts of microorganisms of native grasses are shown in Table 1. Overall, 10^5^ (cfu/g of FM) LAB, 10^7^ (cfu/g of FM) aerobic bacteria, 10^4^–10^5^ (cfu/g of FM) coliform bacteria, 10^3^–10^5^ (cfu/g of FM) yeast, and 10^4^–10^5^ (cfu/g of FM) molds were found, and there was no big difference among the counts of bacteria from all silage samples. After 30 days of ensiling, 10^6^–10^7^ (cfu/g of FM) LAB, 10^3^ (cfu/g of FM) aerobic bacteria, 10^2^–10^4^ (cfu/g of FM) yeasts were found in the native grasses. No coliform bacteria or mold was detected in any silage.

**Table 1:** Microbial population (cfu/g FM) of native grasses.

Grass	Lactic acid bacteria	Aerobic bacteria	Coliform bacteria	Yeasts	Molds
BN					
Material	1.63 × 10^5^	3.07 × 10^7^	5.34 × 10^5^	1.63 × 10^3^	1.21 × 10^5^
Silage	1.00 × 10^7^	2.63 × 10^3^	ND	1.10 × 10^4^	ND
CL					
Material	1.74 × 10^5^	1.83 × 10^7^	2.79 × 10^5^	1.35 × 10^4^	9.89 × 10^4^
Silage	1.00 × 10^7^	3.63 × 10^3^	ND	1.28 × 10^2^	ND
SH					
Material	1.52 × 10^5^	5.34 × 10^7^	2.42 × 10^4^	1.28 × 10^4^	1.34 × 10^4^
Silage	6.17 × 10^7^	2.40 × 10^3^	ND	1.38 × 10^3^	ND
RM					
Material	1.37 × 10^5^	5.34 × 10^7^	3.09 × 10^5^	3.82 × 10^5^	2.31 × 10^5^
Silage	5.01 × 10^6^	1.07 × 10^3^	ND	1.38 × 10^3^	ND
SP					
Material	1.24 × 10^5^	2.34 × 10^7^	8.07 × 10^5^	1.60 × 10^4^	4.92 × 10^5^
Silage	1.07 × 10^6^	1.18 × 10^3^	ND	1.17 × 10^3^	ND

BN, Baical Needgrass; CL, China Leymus; SH, Scabrous Hideseedgrass; RM, Ruthenia Medic; SP, Slenderleaf Pulsatill.

### 3.2. Chemical composition and fermentation quality of native grass silages

The chemical composition and fermentation quality of native grass silages are shown in Tables 2, 3. All silages had low moisture with DM contents of native grasses ranging from 40.57 to 52.17% on an FM basis. The DM contents of CL (50.36% DM) and SP (39.45% DM) silages were lower than those of other treatments (*p* < 0.05) after ensiling. The CP content of RM (fresh 13.51% DM, silage 14.55% DM) was higher than other treatments (*p* < 0.05). The NDF and ADF contents of the native grasses after ensiling were similar, and SP had the lowest NDF (53.26% DM) and ADF (36.05% DM) among the samples. Total WSC content of CL (1.46% DM), SP (1.40% DM) and BN (1.37% DM) silages were higher than RM (1.30% DM) and SH (1.27% DM; *p* < 0.05).

**Table 2:** Fermentation quality of native grasses.

Grass	BN	CL	SH	RM	SP	SEM	*p*-value
pH	4.16 ± 0.05c	4.15 ± 0.09c	4.26 ± 0.07b	4.32 ± 0.03b	4.44 ± 0.07a	0.0294	0.0284
Lactic acid (%FM)	0.84 ± 0.03ab	0.95 ± 0.03a	0.72 ± 0.05bc	0.77 ± 0.03b	0.58 ± 0.08c	0.0469	0.0028
Acetic acid (%FM)	0.38 ± 0.02a	0.42 ± 0.02a	0.36 ± 0.01a	0.38 ± 0.02a	0.28 ± 0.04b	0.0227	0.0186
Butyric acid (%FM)	ND	ND	ND	ND	ND	–	–
Propionic acid (%FM)	ND	ND	ND	ND	ND	–	–
NH_3_-N (%FM)	0.55 ± 0.01b	0.51 ± 0.06b	0.52 ± 0.02b	0.58 ± 0.02b	0.72 ± 0.03a	0.0349	0.0104

^a–c^Means ± SD within rows with different superscript letters differ (*p* < 0.05); Data ± SD are means of three grass samples. BN, Baical Needgrass; CL, China Leymus; SH, Scabrous Hideseedgrass; RM, Ruthenia Medic; SP, Slenderleaf Pulsatilla; FM, fresh matter; ND, not detected.

**Table 3:** Chemical composition of native grasses.

Grass	BN	CL	SH	RM	SP	SEM	*p*-value
DM (%FM)							
Material	51.66 ± 0.51Aa	51.61 ± 0.46Aa	52.17 ± 2.29Aa	42.22 ± 0.24Ab	40.57 ± 0.12Ab	1.0561	<0.0001
Silage	50.94 ± 0.64Aa	50.36 ± 0.29Ba	50.94 ± 0.02Aa	41.90 ± 0.40Ab	39.45 ± 0.05Bc	0.3598	<0.0001
OM (% DM)							
Material	94.79 + 0.01Aa	94.33 ± 0.05Aab	94.11 ± 0.27Ab	94.24 ± 0.07Aab	94.58 ± 0.29Aab	0.1810	0.1312
Silage	93.87 ± 0.39Aab	94.01 ± 0.05Bab	93.57 ± 0.16Ab	94.13 ± 0.01Aab	94.41 ± 0.11Aa	0.1944	0.1041
CP (% DM)							
Material	9.01 ± 0.11Ab	9.65 ± 0.20Ab	8.96 ± 0.20Ab	14.55 ± 0.76Aa	8.72 ± 0.01Ab	0.3643	<0.0001
Silage	8.28 ± 0.45Ab	8.55 ± 0.31Bb	7.94 ± 0.49Ab	13.51 ± 0.51Aa	7.45 ± 0.16Ab	0.4058	<0.0001
EE (% DM)							
Material	2.00 ± 0.16Ab	2.20 ± 0.04Aab	2.13 ± 0.05Ab	2.49 ± 0.14Aa	1.90 ± 0.04Ab	0.1011	0.0172
Silage	1.83 ± 0.03Ab	2.17 ± 0.08Aa	1.83 ± 0.01Bb	2.25 ± 0.09Aa	1.86 ± 0.02Ab	0.0544	0.0004
NDF (% DM)							
Material	67.74 ± 0.78Aa	63.76 ± 2.34Aa	66.20 ± 1.32Aa	64.16 ± 3.28Aa	55.12 ± 2.22Ab	2.1688	0.0167
Silage	63.04 ± 1.17Ba	60.31 ± 2.64Aab	60.54 ± 1.35Bab	62.26 ± 3.37Aa	53.26 ± 3.33Bb	2.5523	0.1295
ADF (% DM)							
Material	45.36 ± 0.63Aa	41.52 ± 0.16Aab	42.71 ± 1.83Aab	43.24 ± 1.69Aa	38.16 ± 1.91Ab	1.4321	0.0519
Silage	42.10 ± 1.21Aa	42.22 ± 1.73Aa	40.80 ± 1.98Aab	43.69 ± 1.49Aa	36.05 ± 1.69Ab	1.5165	0.0410
WSC (% DM)							
Material	2.44 ± 0.02Aa	2.50 ± 0.05Aa	2.19 ± 0.05Abc	2.28 ± 0.03Ab	2.08c ± 0.01Ac	0.0338	<0.0001
Silage	1.37 ± 0.03Babc	1.46 ± 0.05Ba	1.27 ± 0.05Bc	1.30 ± 0.03Bbc	1.40 ± 0.01Bab	0.0364	0.0260

^A–B^Means ± SD within columns with different superscript letters differ (*p* < 0.05); ^a–c^Means ± SD within rows with different superscript letters differ (*p* < 0.05); Data ± SD are means of three grass samples. BN, Baical Needgrass; CL, China Leymus; SH, Scabrous Hideseedgrass; RM, Ruthenia Medic; SP, Slenderleaf Pulsatilla. FM, fresh matter. ND, not detected. DM, dry matter; OM, organic matter; CP, crude protein; EE, ether extract; NDF, neutral detergent fiber; ADF, acid detergent fiber; WSC, water-soluble carbohydrate.

Native grasses significantly affected the pH, lactic acid, and NH_3_-N content (*p* < 0.05). The lowest pH (4.15), NH_3_-N (0.51% FM), and the highest lactic acid (0.95% FM) were observed in CL silage. No butyric acid or propionic acid was detected in any silage, indicating a good fermentation quality. Correlation coefficients (*r*) for the relationship between native grass chemical composition and silage fermentation are shown in Table 4. For pH, a significant negative relationship was observed with WSC content. The lactic acid content was significantly positive with WSC content. The NH_3_-N content was significantly negative with WSC and DM content (*p* < 0.05).

**Table 4:** Correlation coefficients (*r*) for relationships between chemical composition and fermentation silages after 60 days of ensiling.

Variable	LAB	WSC	DM	CP
pH	−0.36 (>0.1)	−0.70 (0.004)**	−0.61 (0.018)*	0.12 (>0.1)
LA	0.30 (>0.1)	0.67 (0.007)**	0.62 (0.013)*	0.10 (>0.1)
NH_3_-N	−0.19 (>0.1)	−0.82 (<0.001)**	−0.70 (0.004)**	−0.02 (>0.1)

LAB, lactic acid bacteria; LA, lactic acid; WSC, water-soluble carbohydrate; DM, dry matter; CP, crude protein. *p < 0.05, **p < 0.01.

### 3.3. Morphological, physiological, and biochemical

The morphological, physiological, and biochemical are shown in Table 5. A total of 54 isolates were collected, of which 48 were considered as LAB by Gram staining and catalase activity. According to the results of physiological and biochemical tests, these isolates were divided into 12 (A-L) groups, and 12 representative strains were used for phylogenetic analysis. Strains in groups A-K were cocci, while group L had rod-shaped bacteria. All isolates were gram-positive and catalase-negative bacteria, growing at temperatures of 5.0 and 10.0, at 3.0 and 6.5% (w/v) NaCl, and at pH 3.5 to 9.0, the 16S rRNA similarity of all isolates was higher than 98%.

**Table 5:** Characteristics of representative strains isolated from natural grasses.

	Group A	Group B	Group C	Group D	Group E	Group F	Group G	Group H	Group I	Group J	Group K	Group L
Characteristic	IAH15	IAH10	IAH45	IAH41	IAH43	IAH26	IAH38	IAH1	IAH44	IAH42	IAH17	IAH14
Shape	Cocci	Cocci	Cocci	Cocci	Cocci	Cocci	Cocci	Cocci	Cocci	Cocci	Cocci	Rod
Grain stain	+	+	+	+	+	+	+	+	+	+	+	+
Catalase	−	−	−	−	−	−	−	−	−	−	−	−
Gas from glucose	+	+	+	+	+	−	−	−	−	−	−	−
Final pH in MRS broth	3.85	3.94	3.90	3.92	3.92	4.52	4.52	3.83	3.95	3.95	3.74	3.52
Fermentation type	Hetero	Hetero	Hetero	Hetero	Hetero	Homo	Homo	Homo	Homo	Homo	Homo	Homo
Option form of lactate	D(−)	D(−)	D(−)	D(−)	D(−)	DL	DL	L(−)	L(−)	L(−)	L(−)	DL
Growth at temperature (°C)												
5.0	+	+	+	+	+	+	+	+	+	+	+	+
10.0	+	+	+	+	+	+	+	+	w	w	+	+
45.0	w	w	w	w	w	w	w	w	w	w	+	+
50.0	−	−	−	−	−	−	−	−	−	−	−	−
Growth in NaCl (%)												
3.0	+	+	+	+	+	+	+	+	+	+	+	+
6.5	w	w	w	+	+	+	+	w	w	w	+	+
Growth at pH												
2.5	−	−	−	−	−	−	−	−	−	−	−	−
3.0	w	w	w	+	+	w	w	w	w	w	+	+
3.5	+	+	+	+	+	+	+	+	+	+	+	+
4.0	+	+	+	+	+	+	+	w	+	+	+	+
7.0	+	+	+	+	+	+	+	+	+	+	+	+
9.0	+	+	+	+	+	+	+	+	+	+	+	+
16S rDNA similarity with each typestrain (%)	99.7%	99.9%	98.9%	100.0%	100.0%	99.0%	99.0%	99.8%	99.7%	99.7%	100.0%	100.0%

+, positive; −, negative; w, weakly positive; MRS, de Man, Rogosa, Sharpe. Hetero-, heterofermentative; Homo, homofermentative.

The carbohydrate fermentation patterns of LAB strains are shown in Table 6. All strains did not produce acid from Glycerol, Erythritol, D-Arabinose, Inositol, Fucose, and L-Arabitol. Group A-E were heterofermentative cocci that formed D (−) lactic acid, producing acid from D-Glucose, D-Fructose, and D-Mannose. Group F and G were homofermentative cocci that formed DL-lactic acid from D-Xylose, Galactose, D-Glucose, D-Fructose, D-Mannose, and Cellobiose. Group H-K were homofermentative cocci that formed L-lactic acid from L-Arabinose, Ribose, D-Xylose, D-Glucose, Esculin, and Cellobiose. Group L were homofermentative rods that formed DL lactic acid from Ribose, D-Xylose, D-Glucose, and Cellobiose.

**Table 6:** API 50 CH (bioMerieux, Tokyo, Japan) characteristics of representative strains isolated from natural grasses.

	Group A	Group B	Group C	Group D	Group E	Group F	Group G	Group H	Group I	Group J	Group K	Group L
Carbohydrate	IAH15	IAH10	IAH45	IAH41	IAH43	IAH26	IAH38	IAH1	IAH44	IAH42	IAH17	IAH14
Glycerol	−	−	−	−	−	−	−	−	−	−	−	−
Erythritol	−	−	−	−	−	−	−	−	−	−	−	−
D-Arabinose	−	−	−	−	−	−	−	−	−	−	−	−
L-Arabinose	−	+	+	+	+	+	+	+	+	+	+	+
Ribose	+	+	+	+	+	+	+	+	+	+	+	+
D-Xylose	w	−	+	+	+	+	+	+	+	+	+	−
L-Xylose	+	−	−	−	−	−	−	−	−	−	−	−
Adonitol	−	−	−	−	−	−	−	+	−	−	−	−
β-Methyl-xyloside	−	−	−	−	−	−	−	+	−	−	−	−
Galactose	+	+	w	+	+	+	+	+	+	+	+	+
D-Glucose	+	+	+	+	+	+	+	+	+	+	+	+
D-Fructose	+	+	+	+	+	+	+	+	+	+	+	+
D-Mannose	+	+	+	+	+	+	+	w	+	+	+	+
L-Sorbose	−	−	−	−	−	−	−	−	−	−	−	w
Rhamnose	−	−	−	w	w	w	w	−	−	−	−	−
Dulcitol	−	+	−	−	−	−	−	−	−	−	−	−
Inositol	−	−	−	−	−	−	−	−	−	−	−	−
Mannitol	−	+	−	−	−	−	−	+	−	−	−	+
Sorbitol	−	−	−	−	−	−	−	+	−	−	−	+
α-Methyl-D-mannoside	−	+	−	−	−	−	−	+	−	−	−	+
α-Methyl-D-glucoside	+	+	+	−	−	−	−	w	−	−	−	−
*N*-Acetyl glucosamine	+	+	+	w	w	+	+	+	+	+	+	+
Amygdalin	+	w	+	+	+	+	+	+	+	+	+	+
Arbutin	+	+	+	+	+	+	+	+	+	+	+	+
Esculin	+	+	+	+	+	+	+	+	+	+	+	+
Salicin	+	+	w	−	−	+	+	+	+	+	+	+
Cellbiose	+	+	+	+	+	+	+	+	+	+	+	+
Maltose	+	+	+	−	−	−	−	+	+	+	+	+
Lactose	+	+	−	−	−	−	−	+	+	+	+	+
Melibiose	+	+	+	−	−	−	−	+	+	+	+	+
Saccharose	+	+	+	−	−	−	−	w	+	+	+	+
Trehalose	+	w	+	+	+	w	w	+	+	+	+	+
Insulin	+	−	−	−	−	−	−	w	−	−	−	−
Melezitose		−	−	−	−	w	w	−	−	−	−	+
D-Raffinose	w	−	−	−	−	−	−	−	−	−	−	+
Starch	−	−	−	−	−	−	−	−	−	−	−	w
Glycogen	−	−	−	−	−	−	−	w	−	−	−	−
Xylitol	−	−	−	−	−	−	−	+	−	−	−	+
β-Gentiobiose	+	w	w	w	w	+	+	−	−	−	−	w
D-Turanose	+	−	−	−	−	−	−	−	−	−	−	+
D-Lyxose	−	−	−	−	−	−	−	−	−	−	−	−
D-Tagatose	−	w	−	+	+	+	+	−	−	−	−	−
D-Fucose	−	−	−	−	−	−	−	−	−	−	−	−
L-Fucose	−	−	−	−	−	−	−	−	−	−	−	−
D-Arabitol	−	−	−	−	−	−	−	−	−	−	−	w
L-Arabitol	−	−	−	−	−	−	−	−	−	−	−	−
Gluconate	−	w	−	−	−	−	−	w	+	+		−
2-K-Gluconate	w	−	−	−	−	−	−	−	−	−	−	−
5-K-Gluconate	−	−	−	−	−	−	−	−	−	−	−	−

+ positive, −, negative; w, weakly positive; IA, Inner Mongolia Agriculture.

### 3.4. Phylogenetic trees of 16S rRNA gene sequence

**Figure 1:**
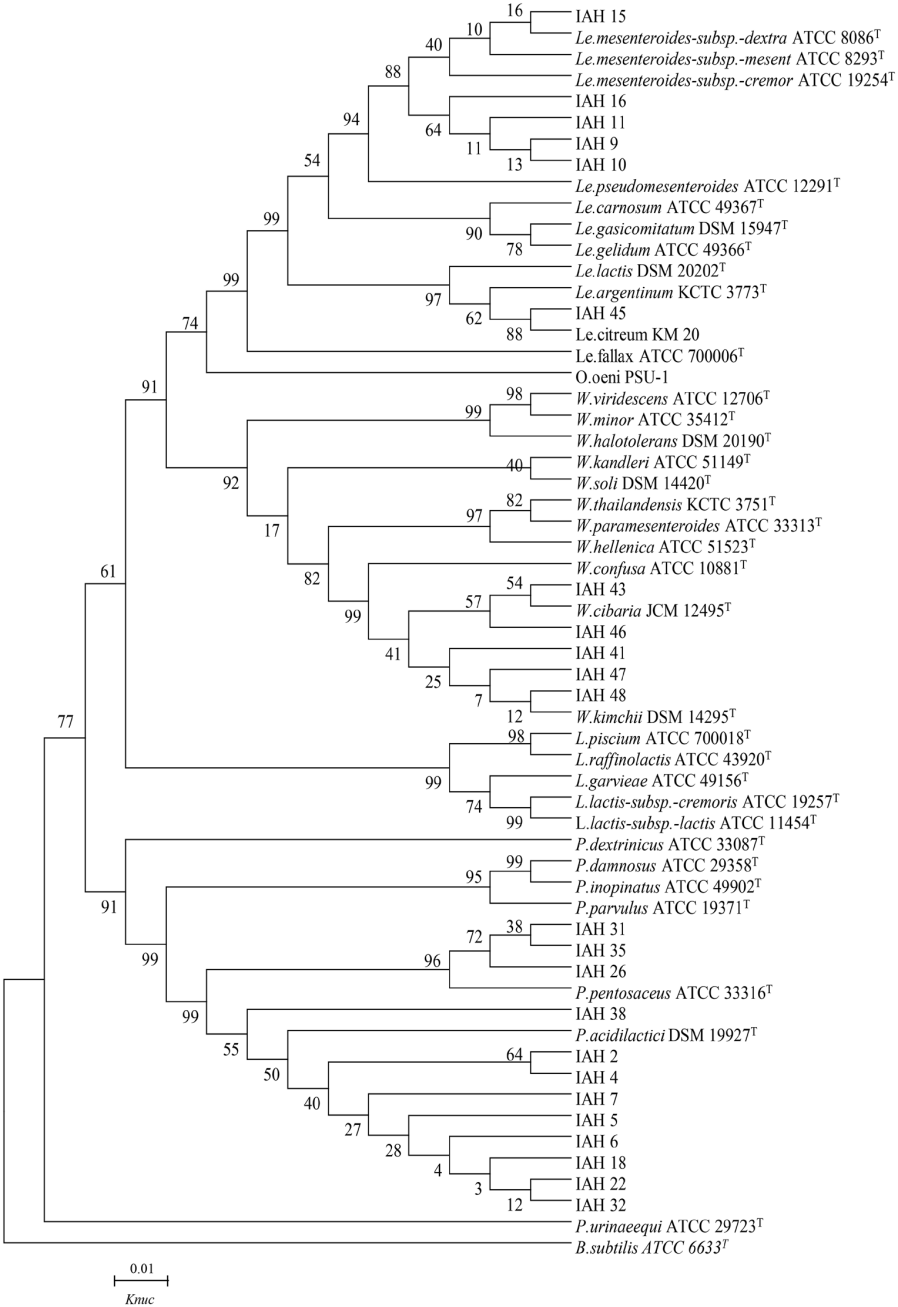
Phylogenetic tree showing the relative position of *Leuconostoc*, *Weissella*, *Lactococcus*, and *Pediococcus* species and representative strains isolated from natural grasses as inferred by the neighbor-joining method of complete 16S rDNA sequences with 1,000 replicates. Reference sequences of type strains from GenBank are used for comparison, *Bacillus subtilis* is used as the outgroup. The width is proportional to terminal branch lengths (1% sequence divergence, shown as a scale bar at lower right). *Le.*, *Leuconostoc*; *W.*, *Weissella*; *L.*, *Lactococcus*; *P.*, *Pediococcus*. *K*nuc, nucleotide substitution rates.

**Figure 2:**
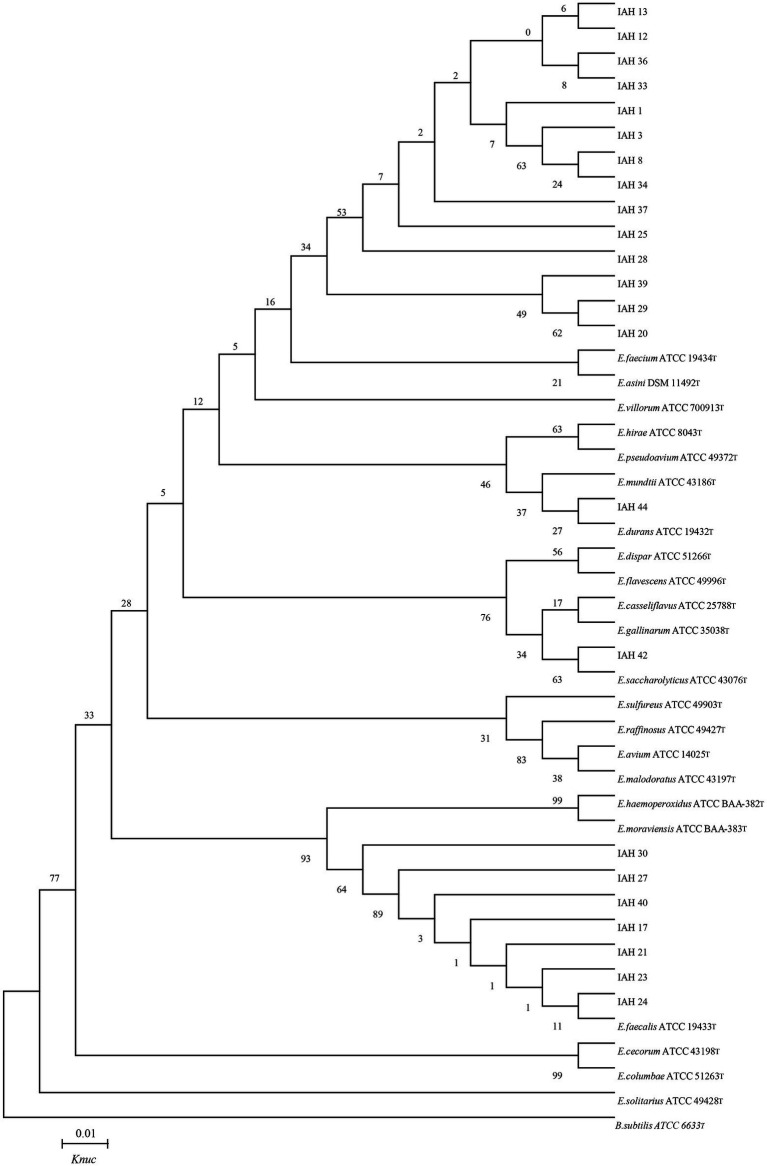
Phylogenetic tree showing the relative position of *Enterococcus* species and representative strains isolated from natural grasses as inferred by the neighbor-joining method of complete 16S rDNA sequences with 1,000 replicates. Reference sequences of type strains from GenBank are used for comparison, *Bacillus subtilis* is used as the outgroup. The width is proportional to terminal branch lengths (1% sequence divergence, shown as a scale bar at lower right). *E., Enterococcus*. *K*nuc, nucleotide substitution rates.

**Figure 3:**
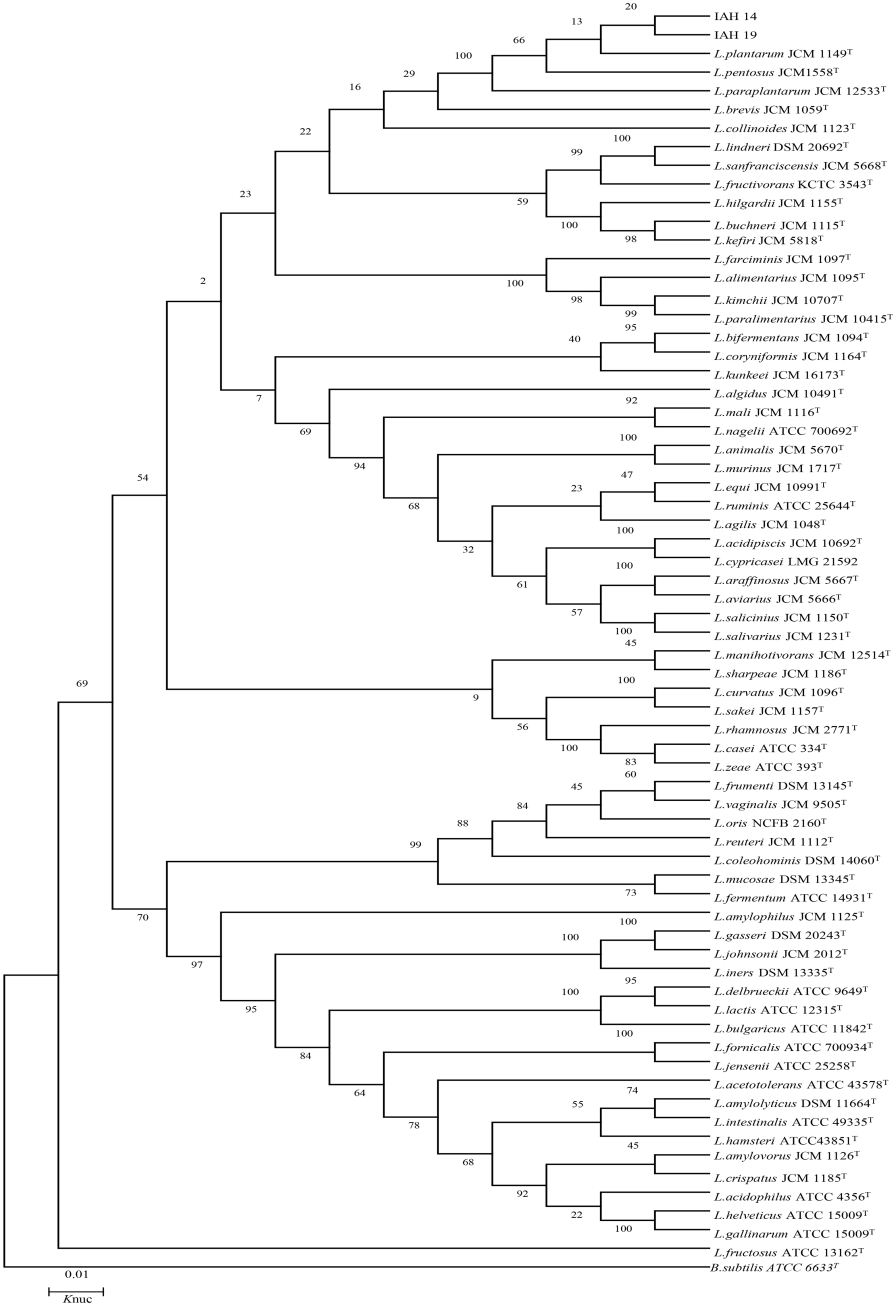
Phylogenetic tree showing the relative position of *Lactiplantibacillus* species and representative strains isolated from natural grasses as inferred by the neighbor-joining method of complete 16S rDNA sequences with 1,000 replicates. Reference sequences of type strains from GenBank are used for comparison, *Bacillus subtilis* is used as the outgroup. The width is proportional to terminal branch lengths (1% sequence divergence, shown as a scale bar at lower right). *L.*, *Lactiplantibacillus*. *K*nuc, nucleotide substitution rates.

The phylogenetic trees of 16S rRNA gene sequences are shown in Figures 1–3. Strains in Groups A-C were placed in the cluster of genera *Leuconostoc (Le.)*, since they were grouped on the tree together with *Le. mesenteroides-subsp.-dextra*, *Le. Pseudomesenteroides*, and *Le. citreum*. Sktrains in Groups D-G were identified as *Weissellla* and *Pediococcus*. Strains in Group D and E were newly described as the newly species *Weissellla cibaria* and *Weissellla kimchii* with similarity exceeding 99.0%. Strains in Group F and G were assigned to *Pediococcus pentosaceus* and *Pediococcus acidilactici.* Strains in H to K were clearly identified as *Enterococcus faecium*, *Enterococcus durans*, *Enterococcus saccharolyticus,* and *Enterococcus faecalis,* Strains in Group L were placed in the cluster of genus *Lactiplantibacillus* on the phylogenetic tree and clearly identified as *Lactiplantibacillus plantarum* by their sequence similarity at 100%, which was only presented in BN and CL silages.

### 3.5. Microbial community of native grass silages

**Figure 4:**
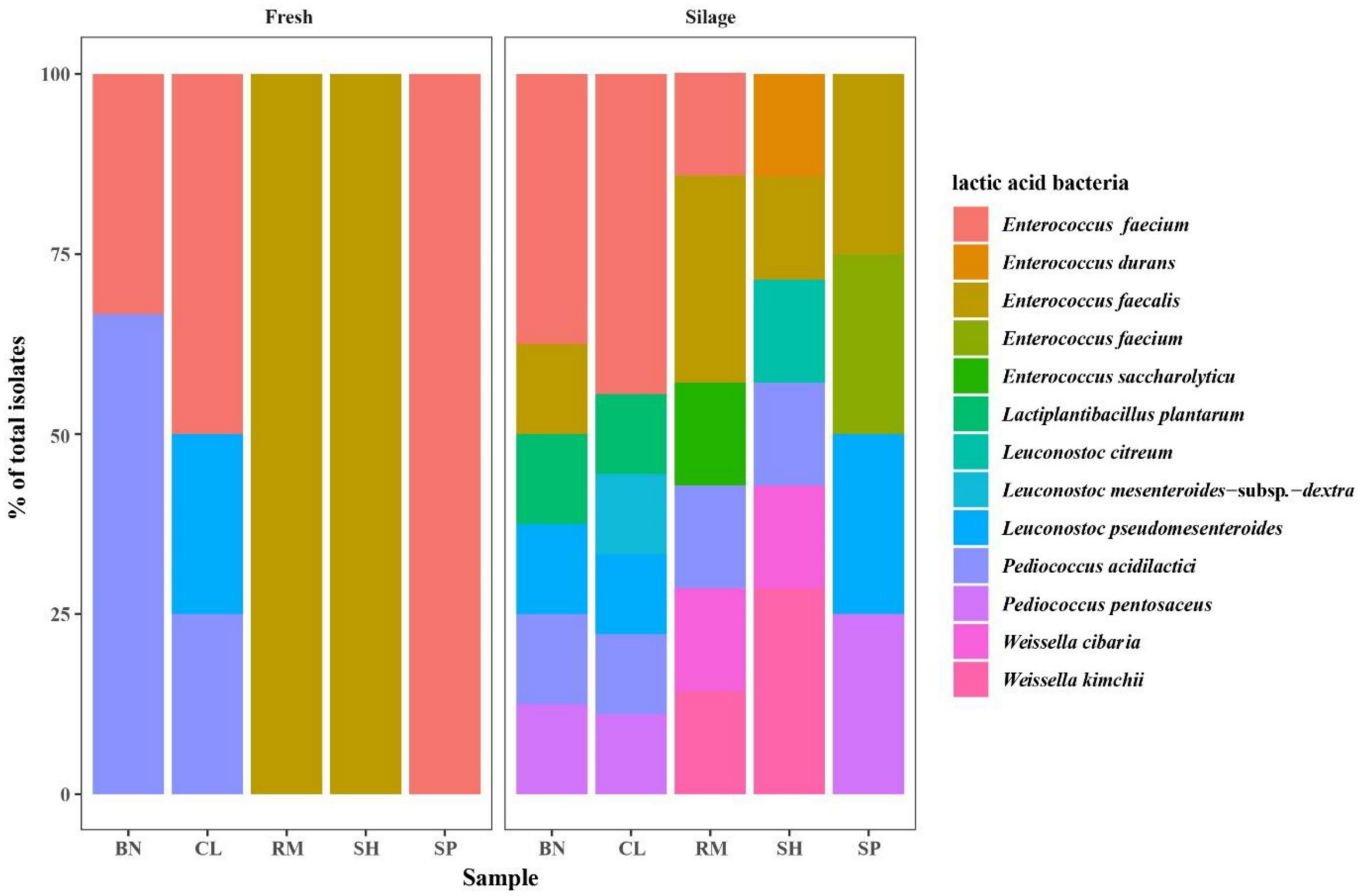
Community of lactic acid bacteria of native grass. BN, Baical Needgrass; CL, China Leymus; SH, Scabrous Hideseedgrass; RM, Ruthenia Medic; SP, Slenderleaf Pulsatilla.

The LAB community of native grass and their silages are shown in Table 7 and Figure 4. The microbial diversity of LAB consisted of 12 species: *Le. mesenteroides*-subsp.-*dextra* (2.08%), *Le. pseudomesenteroides* (8.33%), *Le. citreum* (2.08%), *W. cibaria* (4.17%), *W. kimchii* (6.25%), *P. pentosaceus* (6.25%), *P. acidilactici* (18.75%)*, E. faecium* (29.17%), *E. durans* (2.08%), *E. saccharolyticu* (2.08%), *E. faecalis* (14.58%), and *L. plantarum* (4.17%). Furthermore, most species were homofermentative and accounted for 76.92% of total fermentation. *Pediococcus acidilactici*, *E. faecium*, and *E. faecalis* were the predominant species in native grass. After 30 days of fermentation, the dominant LAB species were *E. faecium* (37.50 to 44.44%) in BN and CL silages, *E. faecalis* (28.57%) in RM silage, and *W. kimchii* (28.57%) in SH silage. The dominance of particular microbes was not detected in other silages.

**Table 7:** The strains and source of LAB isolated from native grasses.

Group	Strain	Isolated sources	Species	% (total 48)
Group A	IAH15(S)	Slenderleaf Pulsatilla	*Le. mesenteroides-subsp.-dextra*	2.08
Group B	IAH9(F), IAH10(F), IAH11(S), IAH16(S)	China Leymus(9, 10, 11), Scabrous Hideseedgrass(16)	*Le. pseudomesenteroides*	8.33
Group C	IAH45(S)	Scabrous Hideseedgrass	*Le. citreum*	2.08
Group D	IAH43(S), IAH46(S)	Ruthenia Medic(43), Scabrous Hideseedgrass(46)	*W. cibaria*	4.17
Group E	IAH41(S), IAH47(S), IAH48(S)	Ruthenia Medic(41), Scabrous Hideseedgrass(47, 48)	*W. kimchii*	6.25
Group F	IAH26(S), IAH31(S), IAH35(S)	Slenderleaf Pulsatilla(26), Bacial Needgrass(31), China Leymus(35)	*P. pentosaceus*	6.25
Group G	IAH2(F), IAH4(F), IAH5(F)(F), IAH6(F), IAH7(F), IAH18(S), IAH22(S), IAH32(S), IAH38(S)	China Leymus(2), Bacial Needgrass(4, 5, 6, 7), Scabrous Hideseedgrass(18), Ruthenia Medic(22), Bacial Needgrass(32), China Leymus(38)	*P. acidilactici*	18.75
Group H	IAH1(F), IAH3(F), IAH8(S), IAH12(F), IAH13(F), IAH20(S), IAH25(S), IAH28(S), IAH29(S), IAH33(S), IAH34(S), IAH36(S), IAH37(S), IAH39(F)	China Leymus(8, 12, 13, 33, 34, 36, 37, 39), Bacial Needgrass(1, 3, 8, 28, 29), Ruthenia Medic(20), Slenderleaf Pulsatilla(25)	*E. faecium*	29.17
Group I	IAH44(S)	Scabrous Hideseedgrass(44)	*E. durans*	2.08
Group J	IAH42(S)	Ruthenia Medic(42)	*E. saccharolyticu*	2.08
Group K	IAH17(S), IAH21(F), IAH23(S), IAH24(S), IAH27(S), IAH30(S), IAH40(F)	Scabrous Hideseedgrass(17,) Ruthenia Medic(21, 23, 24, 40), Slenderleaf Pulsatilla (27), Bacial Needgrass(30)	*E. faecalis*	14.58
Group L	IAH14(S), IAH19(S)	China Leymus(14), Slenderleaf Pulsatilla (19)	*L. plantarum*	4.17

IAH strains were determined by the Grain stain appearance, catalase test, and lactic acid productivity. The representative stains were selected by their different morphological and biochemical characters. Collection site: meadow steppe, Hulunbuir, Inner Mongolia, China (48.27°N, 119.44°E). *Le.*, *Leuconostoc*; *W.*, *Weissella*; *P.*, *Pediococcus*; *E.*, *Enterococcus*; *L.*, *Lactiplantibacillus*.

A spearman correlation heatmap of the lactic acid bacteria and fermentation products at the species level was created to better understand the relationship between fermentation and the microbial community (Figure 4). pH and NH_3_-N were negatively correlated with *L. plantarum*, *E. faecium*, and *Le. mesenteroides-*subsp.*-dextra*., while lactic acid showed the opposite patterns. During ensiling, *E. faecium* used the WSC as a substrate to produce lactic acid and preserve forages, while WSC was significantly positively correlated with *E. faecium*.

## 4. Discussion

The importance of native grass as a feed resource in animal husbandry across China has been increasing. Since the weather has become unpredictable, silage production has become a better method of preservation than hay-making in the Inner Mongolian Plateau for ensuring high quality and long-term storage of these resources (Bu et al., 2021; Li Y. et al., 2022). Therefore, studies on chemical composition, fermentation quality, microbial community, characterization, and identification of lactic acid bacteria related to silage fermentation of native grasses in the Mongolian Plateau are necessary to help create good quality silage and for long-term storage.

DM content is one of the key factors in achieving fermentation quality. In this study, the DM contents of all silages were lower than the fresh native grasses, which is consistent with other studies (Fu et al., 2022). The hydrolysis of organic acid during ensiling also reduced the DM content (Zhao et al., 2018). In this study, during the ensiling, WSC was transformed into organic acid, ethanol, and carbon dioxide by microorganisms, resulting in high DM content that inhibited the growth of *Clostridium* spp., which often produces butyric acid and ammonia nitrogen in silage material (McEniry et al., 2011; Muck et al., 2018; Wang et al., 2018). Overall, the lower CP and WSC and higher NDF and ADF agreed with our previous study in meadow steppes (Hou et al., 2017; Du et al., 2020; You et al., 2021a).

The epiphytic LAB used WSC to produce sufficient lactic acid to reduce pH and inhibit the growth of harmful bacteria (He et al., 2021). The LAB of all native grasses presented more than 10^5^ cfu/g of FM, conforming with our previous study on native grasses (Hou et al., 2017; Ge et al., 2018; You et al., 2021a). The lower LAB in silages may have caused the acid-tolerant aerobic bacteria, assisted in some cases by yeasts, to use some lactic acid and nutrients, which is in agreement with the results of Ge et al. (2018). Coliform bacteria were not detected in all silages because acidification might have inhibited the growth of undesirable bacteria. Zhang et al. (2015, 2016a) reported that the activity of LAB caused rapid acidification by the increased lactic acid content and decreasing pH value, which might explain the suppression of the activity of some microorganisms like coliform bacteria on the *L. chinensis* silage.

Ensiling is a process of complex microbial fermentation, resulting in organic acid accumulation. In this study, the native grass silages were well preserved with lactic acid produced, exceeding 0.58% FM (Ding et al., 2020). pH below 4.20 could result in good fermentation quality and inhibit harmful bacteria (Wang, Y. et al., 2019). In our study, the pH in native grass after 30 days of fermentation was 4.15–4.44, similar to the results by You et al. (2021a). After 30 days of ensiling, the CP content of native grass was decreased, which might have been caused by the degradation of proteins by microorganisms (Du et al., 2022). The change of CP and NH_3_-N contents in SP was the highest out of all silages, showing that there was some undesirable microorganism and accumulation of NH_3_-N during the ensiling of SP, which is consistent with the findings of Ni et al. (2017) that the harmful bacteria *Sphingomonas* elevated NH_3_-N and protein breakdown. Liu et al. (2022) reported that Fungi such as *Aspergillus* and *Monascus* had significant positive correlations with CP and resulted in the decrease of nutrients in meadow steppe.

Fresh native grass had lactic acid bacteria, aerobic bacteria, coliform bacteria, yeasts, and molds, whereas silages contained no coliform bacteria or molds, and lactic acid bacteria was the most abundant microorganism. According to certain studies, prevalent LAB were the main microbial community in forage and grass silages [corn, paddy rice, oat, and Italian grass (Ennahar et al., 2003; Cai et al., 2020; Fu et al., 2022; Li H. et al., 2022)]. Cai (1999) found that epiphytic microbial flora isolated from forages and grasses were species of *Lactiplantibacillus* and *Streptococci*, *Enterococci*, *Lactococci*, *Leuconostocs*, *Pediococci*, and *Weissella* with the predominant LAB being homofermentative L (+) lactic acid-producing cocci. In the present study, a total of 54 isolates were screened and purified. According to the gram-stain and catalase tests, 48 isolates were considered as LAB. All strains were characterized by sugar fermentative assays and were divided into 12 groups, representing five genera. It is difficult to identify the isolates by adopting the phenotypic technique for classifying species (Ni et al., 2015). Hence, based on the 16S rRNA gene sequence, strains were identified as *Lactiplantibacillus* (4.17%), *Weissella* (10.42%), *Leuconostoc* (12.5%), *Pediococcus* (25%), and *Enterococcus* (47.92%).

Generally, the homofermentative LAB *L. plantarum, Enterococcus faecium*, and *Pediococcus acidilactici* are used as commercial inoculants (Ennahar et al., 2003). The epiphytic LAB *cocci* grew vigorously in the early stages of ensiling and created an aerobic environment, but *lactobacilli* played a more important role in long-term fermentation (Cai et al., 1998). It was previously reported that *Lactiplantibacillus* promoted silage quality, with *L. plantarum* being the most prevalent species (Kung et al., 2003; Chen et al., 2014; Da Silva et al., 2014; Xu et al., 2021). However, *Lactiplantibacillus* only accounted for 4.17% of the total LAB microflora in the native grass silages of our study. Zhang and Yu (2017), reported that the LAB species of *L. chinensis* also included *Lactiplantibacillus brevis* and *L. casei*, but we did not observe them. *Lactiplantibacillus plantarum* (IAH14) was only found in *Stipa baicalensis* and *L. chinensis* with lower pH and higher lactic acid than in other native grass silages. This could be because the *L. chinensis* in our study was native, but that in Zhang’s study was cultivated (Zhang et al., 2016a).

Group L was identified as *L. plantarum* or *L. pentoses*. Hammes et al. (2005) reported that these two species are genotypically closely related and show highly similar phenotypes in the same 16S rRNA phylogenetic group with only a 2 bp difference. Nevertheless, the only way to distinguish this was through phylogenetic analysis of sequences of the 16S-23S rRNA intergenic spacer region (ISR) or *rec*A gene sequence comparison (Tannock et al., 1999; Torriani et al., 2001). In this study, all strains were tested for their sugar fermentation profiles and were unambiguously identified as *L. plantarum* by carbohydrate utilization pattern, as it produces acid from melezitose, D-raffinose, and α-methyl-D-mannoside (Spano et al., 2002; Cao et al., 2010), which was different from *L. pentoses*. *L. pentoses* can be distinguished from *L. plantarum* by growing D-xylose and glycerol (Zanoni et al., 1987). Hence, Group I (IAH14 and IAH19) were similar to *L. plantarum*. As expected, Group I could regularly grow at pH 3.5 and weakly at pH 3.0, which showed tolerance to acidic environments. *Enterococcus faecium* and *Pediococcus acidilactici* were dominant species in native grass silages, which is supported by a study where the *Enterococcus* and *Pediococcus* were the most frequently found on the surface of forage crops and food products (Cai, 1999; Fugaban et al., 2021). In fact, numerous studies found that *Enterococcus* and *Pediococcus* are active when pH > 5.0 and dominant in the early stages of ensiling, producing greater lactate concentration for further fermentation. In our study, the *Pediococcus* (Group F and G) and *Enterococcus* (Group H-K) could not grow in a low pH (<4.0) condition, which is in agreement with our previous study (Cai, 1999). The final pH of all silages was >4.0 after 30 days of ensiling, thus, some butyric acid fermentation might have occurred, but did not promote silage fermentation.

**Figure 5:**
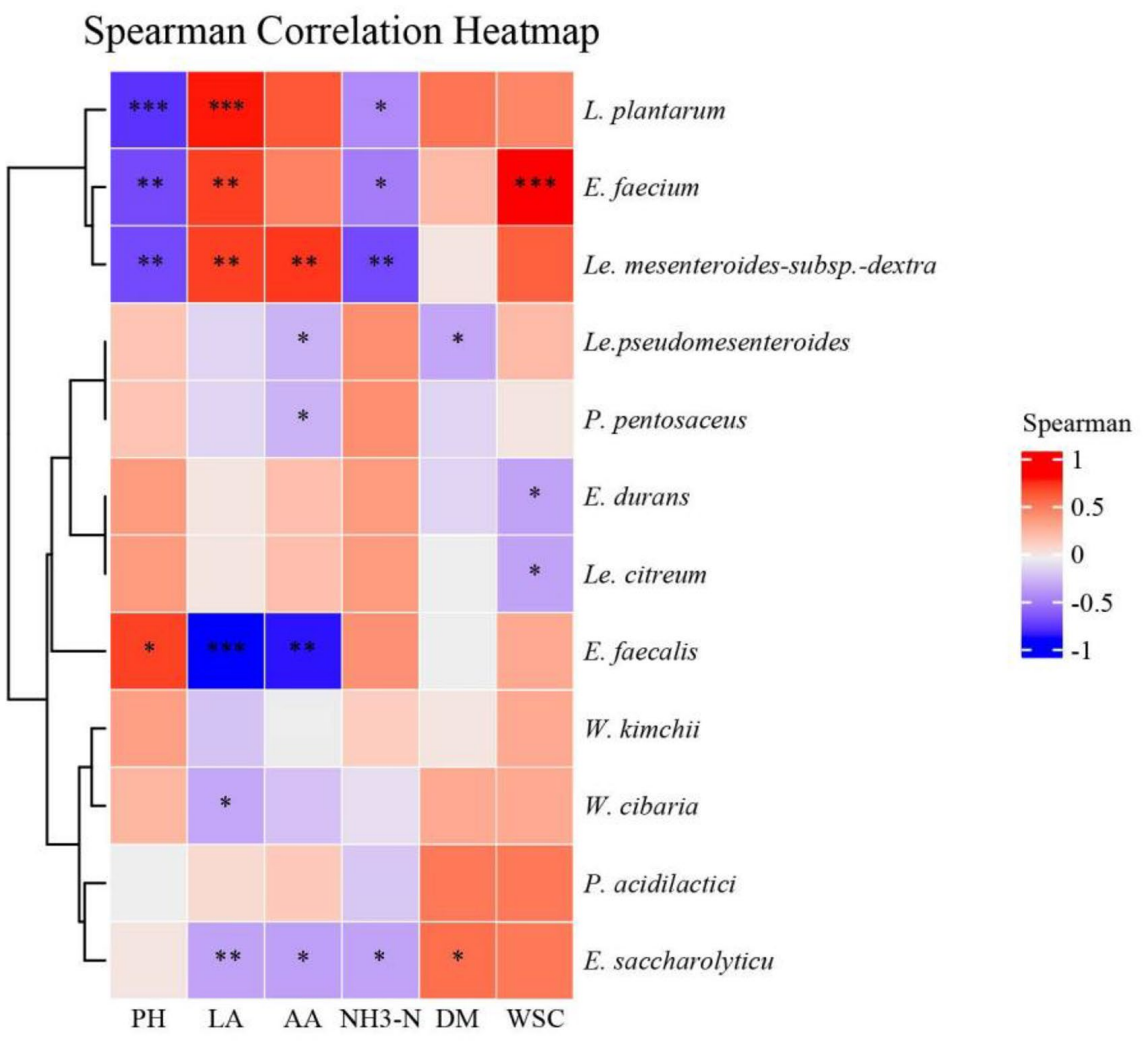
Spearman correlation heatmap of the lactic acid bacteria and fermentation products at species level. NH3-N, ammonia nitrogen; DM, dry matter; WSC, water-soluble carbohydrate; LA, lactic acid; AA, acetic acid. *E., Enterococcus*; *L.*, *Lactiplantibacillus*. *Le.*, *Leuconostoc*; *W.*, *Weissella*; *L.*, *Lactococcus*; *P.*, *Pediococcus*. *p < 0.05, **p < 0.01, ***p < 0.001.

In Figure 5, we found *L. plantarum* was positively correlated with LA and negatively correlated with pH value, which is in accordance with Fang et al. (2022). The LA and the AA contents were negatively correlated with *E. faecalis*, in agreement with a previous study that illustrated the same (Fang et al., 2022). In addition, it was reported that *W. paramesenteroides* was the most abundant species of *Weissela* in forage silage (Libonatti et al., 2019; Wang, S. et al., 2019). However, Chen et al. (2010) found that *W. cibara* was the most abundant species in mulberry farms. This study did not find any *W. paramesenteroides* in native grass. Ennahar and Cai (2004) reported that *W. cibaria* and *W. kimchii* displayed very similar sugar-fermentation patterns and produced the D-form of lactic acid, which indicated that *W. kimchii* was a later heterotypic synonym for *W. cibaria*. Therefore, the strains IAH 43 and IAH 46 from our study, should belong to *W. cibaria.* In a previous study on fresh fruits and vegetables, *W. cibara* strain TM128 decreased infection levels of fungi by 50% (Ennahar and Cai, 2004). Similar work done on durum wheat showed that *W. cibara* strain C21-4 had the strongest antifungal activity, therefore, it is speculated that *W. cibara* may protect the native grass from fungi. This study is the first report of *Weissella* strain in native grass from the Mongolian Plateau. Further studies are necessary to test the effect of *W. cibara* as an additive to maintain native grass silage quality. Additionally, *Weissella* was another dominant genus found in silages throughout fermentation (Guan et al., 2018).

In Inner Mongolia, the studies on native grass silages have been increasing rapidly over the past 5 years (Hou et al., 2017; Ge et al., 2018; Du et al., 2020; You et al., 2021a; Li H. et al., 2022). The present study focused on *L. plantarum* and the silage fermentation of native grass. Future research must take into account this forage’s digestibility for sheep and cows, and how it affects the quality of mutton produced.

## 5. Conclusion

This study revealed that native grass has abundant LAB species and can be well preserved through silage. The strains *P. acidilactici* and *E. faecium* were the most frequently isolated from native grass silages as dominant species. Further studies about the LAB isolated from native grass and their use as additives in silage are needed for developing the potential inoculants for forge silages. Based on the chemical composition and silage fermentation, the Inner Mongolian meadow steppe produced good silage, which can help alleviate the burden of feed shortage in animal husbandry in China.

## Data availability statement

The datasets generated for this study can be found in the NCBI under accession number PRJNA545378.

## Author contributions

MH contributed to methodology, visualization, validation, and data curation and wrote the original draft. ZW contributed toward performing the experiments and funding acquisition. LS contributed significantly to the analysis and manuscript preparation. SW performed the data analyses and wrote the manuscript. YJ and YC contributed to conceptualization and discussions. All authors have read and agreed to publish this version of the manuscript.

## Funding

This work was supported by the Key Laboratory of Forage Cultivation, the Processing and Highly Efficient Utilization of the Ministry of Agriculture, and the Key Laboratory of Grassland Resources of the Ministry of Education. It was funded by the National Key R&D Program of China “Construction of novel additives for total mixed ration and their application” (Grant No. 2022YFE0111000).

## Conflict of interest

The authors declare that the research was conducted in the absence of any commercial or financial relationships that could be construed as a potential conflict of interest.

## Publisher’s note

All claims expressed in this article are solely those of the authors and do not necessarily represent those of their affiliated organizations, or those of the publisher, the editors and the reviewers. Any product that may be evaluated in this article, or claim that may be made by its manufacturer, is not guaranteed or endorsed by the publisher.
